# Investigation on preferably oriented abnormal growth of CdSe nanorods along (0002) plane synthesized by henna leaf extract-mediated green synthesis

**DOI:** 10.1098/rsos.171430

**Published:** 2018-03-14

**Authors:** P. Iyyappa Rajan, J. Judith Vijaya, S. K. Jesudoss, K. Kaviyarasu, Seung-Cheol Lee, L. John Kennedy, R. Jothiramalingam, Hamad A. Al-Lohedan, M. Mahamad Abdullah

**Affiliations:** 1 Indo-Korea Science and Technology Center (IKST), Korea Institute of Science and Technology (KIST), Bangalore, India; 2 Catalysis and Nanomaterials Research Laboratory, Department of Chemistry, Loyola College (Autonomous), Chennai 600 034, India; 3 UNESCO-UNISA Africa Chair in Nanosciences/Nanotechnology Laboratories, College of Graduate Studies, University of South Africa (UNISA), Muckleneuk Ridge, PO Box 392, Pretoria, South Africa; 4 Nanosciences African network (NANOAFNET), Materials Research Group (MRG), iThemba LABS-National Research Foundation (NRF), 1 Old Faure Road, 7129, PO Box 722, Somerset West, Western Cape Province, South Africa; 5 Electronic Materials Research Center, Korea Institute of Science and Technology (KIST), Seoul, Republic of Korea; 6 Materials Division, School of Advanced Sciences, Vellore Institute of Technology (VIT) University, Chennai Campus, Chennai 600 127, India; 7 Chemistry Department, College of Science, King Saud University, Riyadh 11451, Saudi Arabia

**Keywords:** CdSe nanorods, green extract, surface energy, band gap

## Abstract

The theme of this work is to highlight the significance of green plant extracts in the synthesis of nanostructures. In asserting this statement, herein, we report our obtained results on the synthesis of hexagonal CdSe nanorods preferably oriented along (0002) plane through henna leaf extract-mediated reaction along with a discussion about the structural, morphological and optical properties of the synthesized nanorods. The possible mechanism for the synthesis of CdSe nanorods was explored. The formation of nanorods along (0002) plane was confirmed by the relatively high intensity of the (0002) peak in X-ray diffraction pattern. To account for the experimentally realistic condition, we have calculated the surface energies of hexagonal CdSe surface slabs along the low indexed (0002), $\left(10\bar{1}0\right)$ and $\left(11\bar{2}0\right)$ plane surfaces using density functional theory approach and the calculated surface energy value for (0002) surface is 802.7 mJ m^−2^, which is higher than $\left(11\bar{2}0\right)$ and $\left(10\bar{1}0\right)$ surfaces. On realizing the calculated surface energies of these slabs, we determined that the combination of $\left(11\bar{2}0\right)$ and $\left(10\bar{1}0\right)$ planes with lower surface energies will lead to the formation of CdSe nanorods growth along (0002) orientation. Finally, we argue that the design of new greener route for the synthesis of novel functional nanomaterials is highly desired.

## 1. Introduction

An environmentally benign and the rational way of approach towards the synthesis of technologically potent nanostructures is an urgent requirement in the modern scientific research era. The crucial factor lies in the controlling of morphological and structural factors in order to tune up the physical properties of the nanostructured materials [1–5]. Cadmium Selenide (CdSe) semiconductor nanocrystal (NCs) with a moderate bandgap energy (1.74 eV) at 27°C is one among the hot list of materials under both extensive and intensive investigations, owing to its excellent applications in various fields, such as solar cells, light emitting diodes (LEDs), photovoltaic, single-electron transistors, lasers, optoelectronics, sensors and bio-labelling [6,7]. Being a II–VI group semiconductor, CdSe possesses multiple functionalities, such as nonlinear effects, fast optical response, high absorption coefficient, tunable band gap, multi-exciton generation, and extraction of hot electrons [8]. A number of experimental methodologies, such as hydrothermal, solvothermal, inverse micelle, molecular beam epitaxy (MBE) and metal organic chemical vapour deposition (MOCVD) have been proved successful in synthesizing CdSe NCs [9–14]. These methods use toxic reagents and precursors, which need to be replaced by the green synthesis approach. The design and development of green extract-mediated synthesis of technologically potent nanostructures without collapsing the material functionality has been acknowledged widely by the researchers [15,16]. This kind of methodology is always environment-friendly, cheap and involves simple procedures and it has been well proved that the phytochemicals of the plant extracts can act as green fuels for the synthesis of nanostructured materials [17,18]. In addition, we suspect that the use of green extract in the synthesis reaction can attribute the crystal growth in a preferable orientation. The current research paper focuses on the green extract-mediated synthesis of CdSe nanocrystals (NCs) in the form of nanorods oriented along (0002) plane and investigation of its structural, bonding and optical properties. In the hexagonal wurtzite-like structure of CdSe, epitaxial growth will be favoured on (0002) plane under well-controlled synthesis reaction conditions [19] and the morphological, structural and other related properties are strongly influenced by the precursor chemicals and the reaction temperature. In view of these points, combined experimental and theoretical investigations on CdSe NC surfaces were carried out and the results are reported. The surface energy calculations on CdSe (0002−hkil), $\left(10\bar{1}0-\text{hkil}\right)$ and $\left(11\bar{2}0-\text{hkil}\right)$ surfaces were performed based on the density functional theory method.

## 2. Synthesis of CdSe nanorods and its mechanism

The surface-modified CdSe NCs can be achieved by green extract-mediated synthesis reaction. The primary reducing agent used in the current synthesis reaction is henna leaf extract. Fresh henna leaves also termed as hina (*Lawsonia inermis*) were collected from the henna tree for the preparation of leaf broth extract. Twenty-five grams of finely cut and well-washed henna leaves were taken in a beaker containing 100 ml of sterile distilled water. The mixture was then boiled for 1 h and filtered using Whatman filter paper no. 1. Five hundred microlitres of leaf broth was added to 5 ml of cadmium chloride and the selenium which are the source materials for the synthesis reaction. One molar solution of cadmium chloride was prepared by adding 4.58 g of cadmium chloride in 25 ml of water and stirring for 10 min at the temperature range of 10°C to 30°C by using a magnetic stirrer and it is designated as solution A. A selenium solution with a concentration of 0.05 M was prepared by adding 0.0987 g of selenium powder in 10 ml of ethanol and 15 ml of water and stirring for 20 min at 40°C and it is designated as solution B. Then, the solution B is added slowly to the solution A and 5 ml of leaf extract, 2 ml of NaBH_4_ solution was added drop by drop to this mixture and this addition is assisted with continuous stirring. The time of addition of apiin into the metal ion solution was considered as the start of the reaction. Under continuous stirring conditions, after 1 min, the light-yellow colour of CdSe solution turned to brown colour and it indicates the reaction of henna leaves with CdSe solution, which remains stable for more than 2 days without any changes in the absorption spectrum. The synthesized CdSe nanoparticles with the henna leaf-filtered broth extract were kept at 50°C to 60°C for further studies.

The current synthesis reaction predicts that the use of green extract in the synthesis of NCs can significantly contribute to the preferable orientation of crystal growth and influence the NCs morphology. This can be achieved by capping of NCs by the secondary metabolites present in the green extract and finally by the integration of individual NCs along a preferential crystallographic orientation where the phase transformation occurs. The experimental conditions are nearly the same as other synthesis reaction procedures, but the phytochemical content of the green extract dispersed in the suitable solvent will have a strong impact in the growth and morphology of NCs. The metal ions can be reduced to zero valence state by the secondary metabolites of green extract, which occurs through the intermixing of metal salt solution with green extract. Some examples of secondary metabolites are alkaloids, flavonoids, polyphenols and terpenoids. These organic compounds have the tendency to act as the chelating ligands and the metal ions can be easily bound to these compounds. The hydroxyl group is the major binding site with the metal ions and the synthesis mechanism can be summarized into three steps as follows: (i) addition of precursor solution to the green extract will reduce the metal ion to zero valence state and then nucleation occurs; (ii) the nucleated metal NCs interact with each other through spontaneous coalescence, which can be referred to as Ostwald ripening-like reaction; (iii) the final integration of individual NCs along a preferential crystallographic orientation where phase transformation occurs.

## 3. Results and discussion

**Figure 1.**
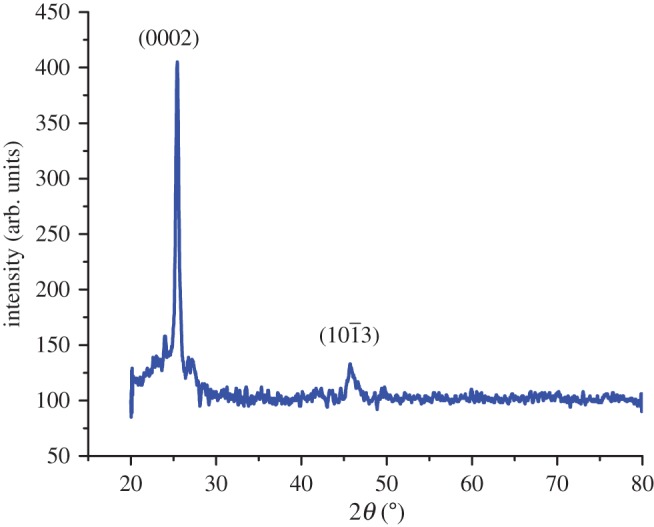
XRD pattern of CdSe nanorods.

X-ray diffraction pattern of CdSe nanorods is shown in figure 1 in which a very high-intensity peak was observed as Bragg's reflection at 2*θ* value of 25.35° accountable to (0002) reflections. The absence of all other peaks with respect to JCPDS card number 08-0459, except a small diffraction peak oriented along (103) at 2*θ* value of 45.78°, was observed in the spectrum. These two peaks were observed with the above JCPDS card number, which belongs to the hexagonal wurtzite structure of CdSe. The plausible reasons for the absence of other Bragg's reflections are due to the following observations: (i) the integration of individual nanorods along a preferential crystallographic orientation, due to the effect of green extract as per the synthesis mechanism explained; (ii) the relatively very high intensity of the (0002) peak allows the CdSe nanorods to grow preferably only along the (0002) direction; (iii) the ultra-thin surface structure of synthesized CdSe nanorods (which can be seen in upcoming SEM results) will lower the intensity of other Bragg's reflections. Also, the high crystalline nature of CdSe was reflected from the high-intensity sharp peak along (0002) direction.

**Figure 2.**
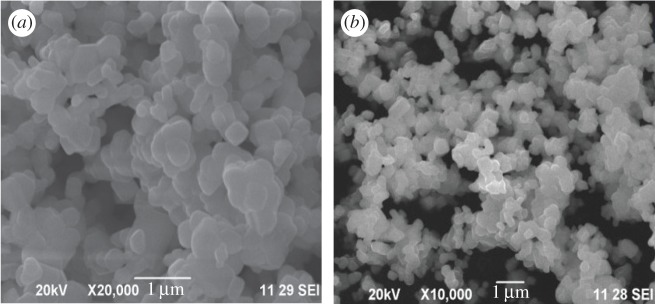
FE-SEM images of CdSe nanorods.

**Figure 3.**
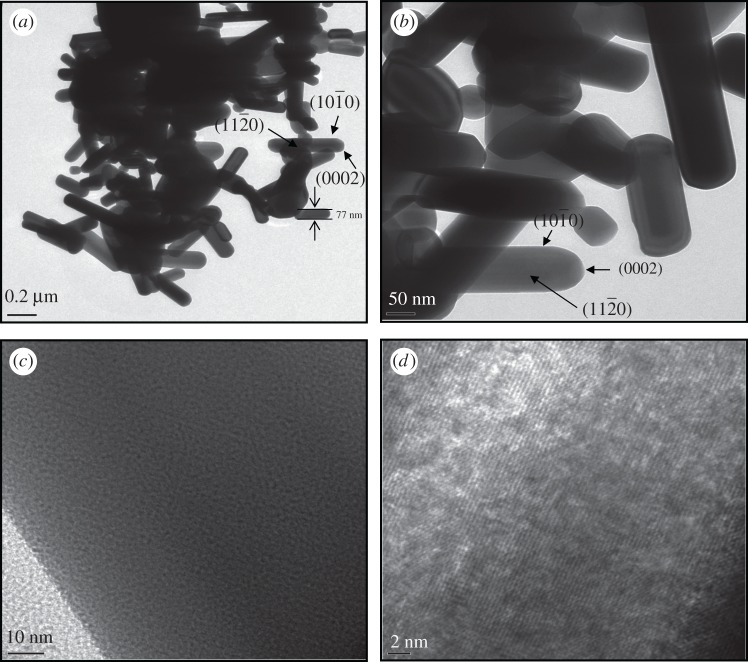
HR-TEM images of CdSe nanorods.

The investigation of morphological characterization of the synthesized CdSe nanorods was performed with a field emission scanning electron microscope (FE-SEM) and a high-resolution transmission electron microscope (HR-TEM) and the respective images captured are displayed in figures 2 and 3. The captured FE-SEM and HR-TEM images show the formation of self-assembled CdSe nanorods along (0002) orientation (c-axis) with the simultaneous formation of $\left(11\bar{2}0\right)$ and $\left(10\bar{1}0\right)$ surfaces, which are more thermodynamically stable than (0002) surface. The nanorods are in hexagonal or distorted hexagonal shape with high agglomeration. The surface of the nanorods displays an ultra-thin surface with an excellent smoothness. The particle size distribution of nanorods is varied relative to each other in the range of nanometres due to the differences in the individual nanorod growth. The high agglomeration of self-assembled CdSe nanorods can be attributed to the following reasons: (i) the large amount of heat energy liberated during the green extract-mediated synthesis reaction will tend to increase the interactions between the nanorods, and (ii) a possible electrostatic interaction between the intercalated CO_3_^2−^ ions and Cd or Se ions, because the intercalation of CO_3_^2−^ ions is feasible during the synthesis reaction using the green extract [17]. The obtained CdSe nanorods after stronger interactions from all the directions are arranged in a random manner as seen in TEM images (figure 3).

**Figure 4.**
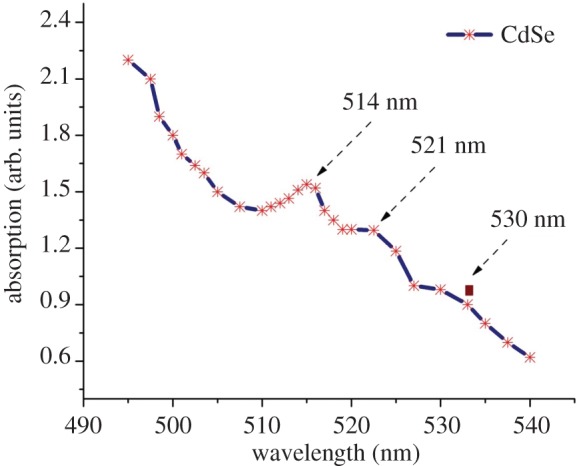
Visible absorption spectrum of CdSe nanorods.

The visible absorption spectrum of the synthesized CdSe nanorods recorded at room temperature is displayed in figure 4. There are broad peaks in the visible absorption curve, which are centred at 514, 521 and 530 nm. The absorption shoulder at 530 nm has an absorption band edge at 540 nm. The calculated optical band gap with respect to this absorption edge (540 nm) is 2.3 eV. Therefore, the results indicated a blue shift of 0.6 eV for CdSe nanorods relative to the bulk CdSe band gap of 1.7 eV at 730 nm [20,21]. Surprisingly, we observed that this is an unusual blue shift still existing in the synthesized nanorods and even the nanorods are comparatively larger than those synthesized in the previously reported work [22]. Therefore, we suspect that the obtained blue-shifted band gap is not due to the quantum confinement effects but it could be due to the preferable orientation growth effects.

As CdSe nanorods are grown larger along (0002) surface, the surface roughness increases from the centre of the nanorod to its edge with an increase in the sharpness at the tip region. In such a more rough surface, the exact width of the nanorods is quite difficult to measure and still we measured the average diameter of the nanorods as seen in the HR-TEM graph (figure 3). Therefore, the excitation peak does not depend on the long axis which grows beyond the confinement regime but depends only on the short axis on the rods which could be a plausible reason for the blue shifting of the excitation peak. In the present case, the nanorod size distribution relatively varies due to the differences in their individual growth. In such cases, the optical band gap measurements cannot be evidence for the quantum confinement effects in nanostructures. As evident from figure 4, the synthesized CdSe nanorods exhibit larger intensity of absorption at the visible region of electromagnetic spectrum, which can be accountable to the strong scattering in this region. Owing to this scattering effect, the optical path tends to increase and the stronger absorption will result in higher incident photon-to-electron conversion efficiency for the operation of photovoltaic solar cells [7,23,24].

**Figure 5.**
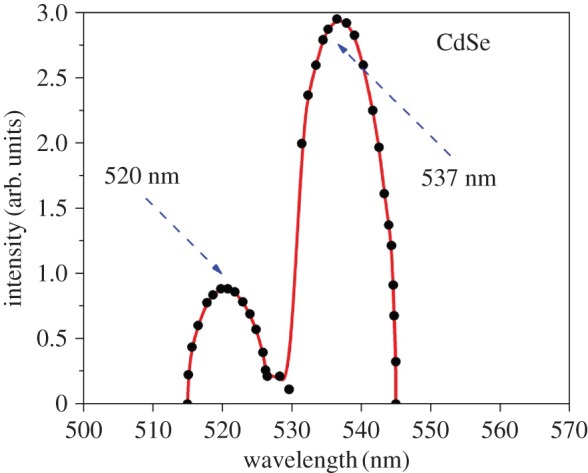
Photoluminescence spectrum of CdSe nanorods at an excitation of 480 nm.

Figure 5 displays the photoluminescence emission spectrum of the synthesized CdSe nanorods recorded with an excitation wavelength of 480 nm. Two emission maximum peaks were observed at 520 and 537 nm. The peaks are strong with narrow band edge emission in the visible region of the electromagnetic spectrum. Also, the emission of surface is visible with major humps in the spectrum, revealing the existence of larger surface area.

**Figure 6.**
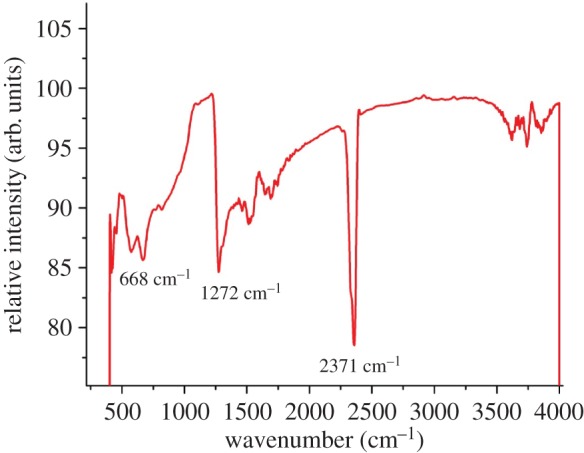
FT-IR spectrum of CdSe nanorods.

The FTIR spectrum of the synthesized CdSe nanorods confirms the Cd-Se stretching vibration at 712 cm^−1^ as displayed in figure 6. Two more IR vibrations were observed at 1272 and 2371 cm^−1^. The band at 1272 cm^−1^ can be accounted to the weaker adsorption of CO_2_ molecules present in the atmosphere during the sampling process [25].

## 4. Density functional theory-based investigation on CdSe hexagonal wurtzite surfaces

To understand the experimental results, we have calculated the surface energies of three low-indexed surfaces along (0002), $\left(10\bar{1}0\right)$ and $\left(11\bar{2}0\right)$ orientations of CdSe hexagonal wurtzite structure. The surface slabs along (0002), $\left(10\bar{1}0\right)$ and $\left(11\bar{2}0\right)$ orientations were modelled as supercells containing 32 atoms having a thickness of eight atomic layers. All the simulations were performed based on the density functional theory code as implemented in Vienna *ab initio* Simulation package (VASP 5.3.5) [26,27]. The representation of electron–ionic core interactions was accounted for by the projector-augmented wave (PAW) method [27,28]. The exchange-correlation effects of the electrons were treated within the Perdew, Burke and Ernzerhof (PBE) method as a revision of generalized gradient approximation (GGA) [29]. A vacuum spacing of 12 Å was applied between the surface slabs throughout the calculations in order to avoid any spurious interactions between the surfaces. The modelled surface slabs were subjected to the ionic relaxation using the conjugate gradient algorithm method [30] and the relaxation was carried out for the three layers of top and bottom of the surface slabs by fixing the middle two layers as bulk positions. A plane wave kinetic energy cut-off of 500.00 eV was used and the Brillouin zone integrations were performed using Gamma-centred *k*-mesh of 4 × 9 × 1, 4 × 5 × 1 and 2 × 4 × 2 for (0002), $\left(10\bar{1}0\right)$ and $\left(11\bar{2}0\right)$ surface slabs, respectively. The total energy convergence for the ionic iterations was 10^−6^ eV, and the ionic iterations were stopped when the interatomic Hellmann–Feynman forces per atom became less than 10^−3^ eV Å^−1^. The dipole correction was applied perpendicular to the surface slabs during the relaxation.

The calculation of surface energy (*γ*_surface_) provides a good knowledge about the surface stability. The surface energy is known to be an excess energy exhibited by the atoms present in the surface of NCs [31]. To calculate the surface energy of CdSe hexagonal surface slabs, we have also relaxed the bulk CdSe hexagonal supercell and the formula for calculating surface energy (*γ*_surface_) is given below.

4.1 $$\gamma_{\mathrm{surface}}=\frac{E_{\mathrm{surface}}-E_{\mathrm{bulk}}}{2A},$$

where, *E*_surface_ is the total energy of CdSe hexagonal surface slab, *E*_bulk_ is the total energy of CdSe hexagonal bulk supercell, *A* is the surface area and *N* is the number of atoms in the bulk supercell structure.

The calculated surface energies (*γ*_surface_) of the CdSe hexagonal surface slabs oriented along (0002), $\left(10\bar{1}0\right)$ and $\left(11\bar{2}0\right)$ planes are 0.8027 J m^−2^ (802.7 mJ m^−2^), 0.2708 J m^−2^ (270.8 mJ m^−2^) and 0.2371 J m^−2^ (237.1 mJ m^−2^), respectively. We have made a comparison of our calculated surface energies with other known reports [32–34] as shown in table 1 and our calculated data show that the surface energy calculated along (0002) plane is higher than those of $\left(11\bar{2}0\right)$ and $\left(10\bar{1}0\right)$ planes. Therefore, we expect that the roughening transition temperature of (0002) surface is lower than those of $\left(11\bar{2}0\right)$ and $\left(10\bar{1}0\right)$ surfaces and, from the FE-SEM and TEM results, one can find the flat surfaces which correspond to $\left(11\bar{2}0\right)$ and $\left(10\bar{1}0\right)$ surfaces and the rough surface that corresponds to (0002) direction. In addition, the lower surface energies of $\left(11\bar{2}0\right)$ and $\left(10\bar{1}0\right)$ surfaces predict the formation of $\left(11\bar{2}0\right)$ and $\left(10\bar{1}0\right)$ surfaces and are thermodynamically more favoured when there is a growth along (0002) orientation. Ultimately, the formation of CdSe hexagonal nanorods along (0002) orientation also results in the combination of $\left(11\bar{2}0\right)$ and $\left(10\bar{1}0\right)$ surfaces. To get the accurate band gaps, we employed the tetrahedron method with Blochl corrections and the calculated band gaps for $\left(11\bar{2}0\right)$ and $\left(10\bar{1}0\right)$ surfaces are 1.085 and 0.782 eV respectively, which are found to be higher than that of the bulk CdSe (0.558 eV) and in turn it confirms the existence of the blue shift from bulk to surfaces as observed in our experiments. It should be noted that the band gap obtained from GGA-PBE calculation could underestimate the experimental values. However, the application of advanced methods, such as hybrid functional, to surfaces is questionable [35]. In addition, the computational cost is very high for hybrid calculations compared to GGA-PBE calculations. To obtain the consistent picture of the band gap, we have used the conventional GGA-PBE method.

**Table 1.** Comparison of surface energies calculated for different surfaces of hexagonal CdSe system.

surface of hexagonal CdSe	surface energy (mJ m^−2^)
current work GGA-PBE	
(0002)	802.7
$\left(10\bar{1}0\right)$	270.8
$\left(11\bar{2}0\right)$	237.1
GGA-PW91 (ref. [32])	
$\left(10\bar{1}0\right)$	245.13
$\left(11\bar{2}0\right)$	232.32
GGA-PW91 (ref. [33])	
$\left(10\bar{1}0\right)$	380
$\left(11\bar{2}0\right)$	270
LDA-CAPZ (ref. [33])	
$\left(10\bar{1}0\right)$	360
$\left(11\bar{2}0\right)$	400
SCC-DFTB (ref. [34])	
$\left(10\bar{1}0\right)$	390
$\left(11\bar{2}0\right)$	400
PP-PBE (ref. [34])	
$\left(10\bar{1}0\right)$	340
$\left(11\bar{2}0\right)$	280

## 5. Conclusion

The current work investigated the structural, morphological and optical properties of hexagonal CdSe nanorods preferably oriented along (0002) plane synthesized by henna leaf green extract-mediated reaction. We have also explored the possible mechanism for the synthesis of CdSe nanorods. The calculated surface energy (*γ*_surface_) value for the CdSe hexagonal surface slab oriented along (0002) plane is relatively very high (0.8027 J m^−2^) and therefore, the growth along (0002) orientation in hexagonal CdSe can be experimentally feasible under the suitable reaction conditions like the green extract-mediated synthesis reaction as presented in this work. The combination of $\left(11\bar{2}0\right)$ and $\left(10\bar{1}0\right)$ planes with the lower surface energies has resulted in the formation of CdSe nanorods along (0002) orientation. An unusual blue-shifted band gap was observed due to the preferable orientation growth effects of nanorods. Finally, we conclude that the design of a new greener route for the synthesis of novel functional nanomaterials along with the theoretical evidence is highly desired.

## Data accessibility

All the data were included sufficiently as figures, plots and tables within the body of manuscript.
