# Correction to ‘Investigation on preferably oriented abnormal growth of CdSe nanorods along (0002) plane synthesized by henna leaf extract-mediated green synthesis’

**DOI:** 10.1098/rsos.180819

**Published:** 2018-06-20

**Authors:** P. Iyyappa Rajan, J. Judith Vijaya, S. K. Jesudoss, K. Kaviyarasu, Seung-Cheol Lee, L. John Kennedy, R. Jothiramalingam, Hamad A. Al-Lohedan, Mahmood M. S. Abdullah

*R. Soc. open sci.* **5**, 171430. (Published online 14 March 2018). (doi:10.1098/rsos.171430)

In the published paper, the last author's name is presented incorrectly. The last author's name is Mahmood M. S. Abdullah.

